# Vascular Inflammation and Endothelial Dysfunction in Experimental Hypertension

**DOI:** 10.4061/2011/281240

**Published:** 2011-09-11

**Authors:** Carmine Savoia, Lidia Sada, Luigi Zezza, Lorenzo Pucci, Francesco Maria Lauri, Alberto Befani, Alessandro Alonzo, Massimo Volpe

**Affiliations:** ^1^Cardiology Unit, Clinical and Molecular Medicine Department, Sant'Andrea Hospital, Sapienza University of Rome, Via di Grottarossa 1037/1039, 00189 Rome, Italy; ^2^IRCCS Neuromed, Polo Molisano University of Rome La Sapienza, Pozzilli, Italy

## Abstract

Essential hypertension is characterized by increased peripheral vascular resistance to blood flow. The endothelium is a crucial regulator of vascular tone. Its function is impaired in patients with hypertension, with reduced vasodilation, increased vascular tone associated with a proinflammatory and prothrombotic state. Low-grade inflammation localized in vascular tissue is therefore recognized as an important contributor to the pathophysiology of hypertension, to the initiation and progression of atherosclerosis as well as to the development of cardiovascular diseases.

## 1. Introduction

Vascular remodeling and increased peripheral vascular resistance to blood flow are common features of essential hypertension. Resistance arteries may play an important role in the development [1] and may contribute to the complications of hypertension [2]. Small decrease in the lumen of the small arteries significantly increases resistance to blood flow. Vascular tone is regulated by the endothelium, which may affect vascular function and remodeling. Endothelium is the active inner monolayer of the blood vessels, forming an interface between circulating blood and the vessel wall. It represents the largest organ in the body and plays a critical role in vascular homeostasis. Endothelial cells regulate vascular tone by releasing various contracting and relaxing factors including nitric oxide (NO), arachidonic acid metabolites, reactive oxygen species (ROS), and vasoactive peptides. Therefore, the endothelium actively regulates vascular tone and permeability, the balance between coagulation and fibrinolysis, the inflammatory activity as well as cell proliferation. Endothelial dysfunction is characterized by impaired vasomotor response (reduced vasodilation and increased endothelium-dependent contraction), cell proliferation, platelet activation, vascular permeability, and a proinflammatory and prothrombotic phenotype, including leucocyte-endothelial interactions that participate in vascular inflammation and increased adhesion and aggregation of platelets [3]. 

Endothelial progenitor cells (EPCs), a bone-marrow-derived population of cells which can develop into competent mature endothelial cells [4], are seen as an important determinant of endothelial function. Decreased EPCs number is associated with arterial stiffness [4] and decreased endothelial function [5]. In this regard, it has been shown that circulating EPCs are significantly reduced in hypertensive type 2 diabetic patients [4] and in salt-loaded hypertensive rats [5]. 

Endothelial dysfunction occurs in association with several cardiovascular risk factors. Hypercholesterolemia, hypertension, and insulin resistance contribute to endothelial dysfunction and inflammation in the vascular wall, as well as to increased lipoprotein oxidation, smooth muscle cell proliferation, extracellular matrix deposition, cell adhesion, and thrombus formation [6–8]. Thus, endothelial dysfunction may be involved in the initiation of vascular inflammation, in the development of vascular remodeling, it is an early determinant in the progression to atherosclerosis, and it is independently associated with increased cardiovascular risk [9–12]. 

Endothelial dysfunction promotes vascular inflammation by inducing the production of vasoconstrictor agents, adhesion molecules, and growth factors including angiotensin II (Ang II) and endothelin 1 [6, 8]. Ang II, one of the final products of the renin-angiotensin system (RAS), is actively involved in the pathophysiology of hypertension [13]. It may be responsible for triggering endothelial dysfunction and vascular inflammation by inducing oxidative stress, resulting in upregulation of inflammatory mediators and cell-growth.

Low-grade inflammation in the vascular wall is an important contributor to the pathophysiology of hypertension [14], atherosclerosis, and the development of cardiovascular disease (CVD) [11, 15]. Patients with CVD present with increased expression and plasma concentration of inflammatory markers and mediators [16, 17]. In particular, increased plasma levels of TNF-a (tumour necrosis factor-a), IL (interleukin)-6, as well as the adhesion molecules ICAM-1 (intercellular adhesion molecule-1), VCAM-1 (vascular cell adhesion molecule-1), and E-selectin, as well as vWF (von Willebrand factor) and CRP (C-reactive protein), have been demonstrated [18–20] in hypertensive subjects. Thus inflammation is a central mechanism contributing to the progression of CVD, and may be involved in the triggering of myocardial and cerebrovascular ischemia [8, 21].

In this paper we discuss the role of the low-grade inflammation in the vascular pathology in experimental hypertension.

## 2. Low-Grade Inflammation and Endothelial Dysfunction in Vascular Pathology

Blood pressure itself or RAS activation [16] may induce the inflammatory process, which participates to vascular remodeling and may contribute to accelerated vascular damage in aging and CVD. Endothelial dysfunction is an early determinant in the development of hypertension, in the progression to atherosclerosis and is independently associated with increased cardiovascular risk [9]. Essential hypertension is characterized by increased peripheral vascular resistance to blood flow, which occurs mostly as a result of energy dissipation in small resistance arteries, particularly in younger individuals. Enhanced constriction of resistance arteries may increase peripheral resistance in hypertension by reducing lumen diameter [22]. Endothelial dysfunction may participate to the increased vascular tone in hypertension [10], with reduced vasodilation associated with a proinflammatory and prothrombotic state. Moreover, in hypertension, resistance arteries undergo vascular remodeling (reduced lumen with increased media width) that may be structural, mechanical, or functional. Extracellular matrix deposition and inflammation are critically involved in vascular remodeling. With chronic vasoconstriction (due to increased myogenic tone, RAS activation, catecholamines, and growth factors production), vessels may become embedded in the remodeled extracellular matrix and may not return to their vasodilated state [23]. Chronically elevated blood pressure and stretch induce complex signal transduction cascades leading to vascular remodeling that contributes not only to elevation of blood pressure but also to hypertensive complications [24]. Arterial hypertension as well as aging and other cardiovascular risk factors may also increase arterial stiffness in large conduit arteries, which is associated with both target organ damage and increased risk for cardiovascular morbidity and mortality [25]. Inflammation of large arteries exerts its effects in part by contributing to endothelial dysfunction and by increasing vascular stiffness, in the presence or absence of hypertension.

Inflammation contributes to vascular remodeling promoting cell growth and proliferation of vascular smooth muscle cells (VSMCs). This is supported by the increased expression in the vascular wall of adhesion molecules and ligands, leukocyte extravasation, increased oxidative stress, cytokine production, activation of immune cells and pro-inflammatory signaling pathways. Greater expression of adhesion molecules (VCAM-1, ICAM-1) on the endothelial cell membrane, accumulation of monocyte/macrophages, dendritic cells, natural killer cells, and B and T lymphocytes participate to the inflammatory response in the vascular wall [26]. Patients with CVD present increased expression and plasma concentration of inflammatory markers and mediators [17, 21, 27]. High levels of inflammatory mediators (IL-6, ICAM-1, and CRP) may be independent risk factors for the development of hypertension [18, 19]. Moreover they have been associated with increased risk of diabetes [20] and CVD. In particular CRP measured with a high-sensitivity assay (hsCRP) has been related to insulin resistance, systolic blood pressure, pulse pressure (PP, an index of arterial stiffness) [28, 29], and to markers of endothelial dysfunction including plasma levels of vWF, tissue plasminogen activator and cellular fibronectin [30]. Plasma hsCRP level is a strong predictor of ischemic cardiovascular events in patients with stable or unstable angina; it correlates with vulnerable plaques, and it may as well predict cardiovascular events among apparently healthy subjects [31–33]. 

Innate immunity may play a role in the mechanisms that contribute to the low-grade inflammatory response in hypertension. In mice deficient in vascular macrophages (due to a mutation in the mCSF gene) Ang II and deoxycorticosterone acetate-(DOCA-) salt were not able to induce hypertension or vascular remodeling [14]. Moreover, mice deficient in T and B lymphocytes presented blunted hypertensive response to Ang II and DOCA-salt [34]. Reduced vascular remodeling in response to Ang II was also observed [34]. The lack of response to Ang II was corrected by effector T cell but not by B lymphocyte adoptive transfer. Moreover, the central and pressor effects of Ang II are critical for T-cell activation and the development of vascular inflammation [35]. T lymphocytes may participate in hypertension and peripheral inflammation possibly by the increased production of oxidative stress [36]. A role of T regulatory (Treg) lymphocytes in blood pressure regulation and vascular inflammation has also been described. It has been recently shown that Treg adoptive transfer lowered blood pressure and protected from vascular remodeling in mice infused with either Ang II or aldosterone. It has been recently showed that adoptive immunity may be enhanced as a result of a genetic predisposition with loci on chromosome 2 (which carries many pro-inflammatory genes) [37] in a consomic strain of rats (SSBN2), which contains the genetic background of hypertensive Dahl salt-sensitive rats and chromosome 2 from Brown-Norway normotensive rats. The presence of the normotensive chromosome 2 was associated with upregulation of Treg markers (CD8+ and CD4+ lymphocytes which were CD25+). Those markers were depressed in the Dahl salt-sensitive rats. Enhanced Treg and increased expression of the transcription factor Foxp3 (a marker of Treg) as well as IL-10 and TGF (transforming growth factor)-beta production (produced by Treg) were found in consomic rats and the opposite in Dahl rats. The potential protective role of Treg in cardiovascular disease is further supported by the evidence that adoptive transfer of Treg cells ameliorated cardiac damage and improved eutrophic remodeling in Ang II-infused mice, independently of blood-pressure-lowering effects [27]. This suggests a role of Treg in the pathogenesis of blood-pressure-induced cardiovascular remodeling. Hence, different subsets of T lymphocytes may be involved in the mechanisms leading to the inflammatory response described in cardiac and metabolic diseases when an imbalance exists between the pro-inflammatory Th1, Th2, and Th17 and the antiinflammatory Treg subsets [27].

## 3. Role of Renin-Angiotensin System in Vascular Damage

Ang II plays a key role in the pathophysiology of hypertension [24] and CVD by inducing vascular remodeling and injury. Several mechanisms may be activated by Ang II such as endothelial dysfunction, vasoconstriction, cell growth, oxidative stress, and inflammation. Ang II stimulates cell growth by inducing hyperplasia and hypertrophy of VSMCs from resistance arteries of patients with essential hypertension and from small arteries of hypertensive rats. 

In hypertension, Ang II may enhance basal superoxide production in the vasculature by activation of reduced NADPH (Nicotinamide Adenine Dinucleotide Phosphate) oxidase and expression of its subunits via cSrc, PKC (Protein Kinase C), PLA_2_ (Phospholipase A_2_), and PLD (Phospholipase D) pathways [38–41]. These processes may contribute to increased activation of NADPH oxidase and, in turn, to enhanced oxidative stress in the vascular wall [10]. NADPH oxidase is the major source of ROS in the vascular wall. It is expressed in endothelial cells, VSMCs, fibroblasts, and monocytes/macrophages [42, 43]. Increased Ang II-induced ROS generation is involved in the process of vascular remodeling. This occurs by VSMC proliferation and hypertrophy, collagen deposition, and by modulating cytokine release and pro-inflammatory transcription factors. ROS activate multiple signaling molecules including mitogen-activated protein kinases (MAP-kinases); nonreceptor tyrosine kinases such as Src, JAK-2 (Janus Kinase 2), STAT (Signal Transducer and Activator of Transcription), p21Ras, Pyk-2 (Proline-rich Tyrosine Kinase 2), and Akt; receptor tyrosine kinases (EGFR (Epidermal Growth Factor Receptor), IGFR (Insulin-like Growth Factor Receptor 1), and PDGFR (Platelet-Derived Growth Factor Receptor); protein tyrosine phosphatases and redox-sensitive transcriptor factors (Nuclear Factor (NF)-*κ*B, Activator Protein 1 (AP)-1 and Hypoxia-inducible factor 1 (HIF-1)) [42, 44]. Thus, ROS act as signaling molecules, modulating vascular tone and structural changes in the vasculature [39] and participate in the development and progression of atherosclerosis [44]. In addition, ROS reduce the vascular bioavailability of NO [45, 46] which is associated with impaired endothelium-dependent vascular relaxation and increased vascular contractile responses. Moreover this is also associated to structural changes in the wall of small arteries and to the increased peripheral vascular resistance [47]. Ang II-induced ROS production increases ICAM-1 expression, macrophage infiltration, monocyte chemotactic protein (MCP)-1 production, and vascular hypertrophy, independently of blood pressure elevation [48]. Furthermore Ang II stimulates the production of E-selectin through redox-dependent pathways [48] and PAI-1, which contributes to a prothrombotic state as well as to atherosclerotic plaque rupture. Moreover, macrophages which infiltrate the adventitia or the media of blood vessels may generate oxidative stress by NADPH oxidase [48, 49]. 

Ang II-induced growth and profibrotic effects are partially modulated by endogenous production of mitogenic factors (including TGF-*β*, PDGF (platelet-derived growth factor), EGF (epidermal growth factor), IGF-1 (insulin-like growth factor 1) and endothelin-1) [50, 51]. In particular TGF-*β* is produced by macrophages, lymphocytes, fibroblasts, VSMCs and is overexpressed in many cardiovascular and renal disorders associated with activation of the RAS. TGF-*β* increases extracellular matrix biosynthesis, downregulates matrix degradative enzymes, and influences integrin receptors [52, 53]. p38MAP kinase and connective tissue growth factor are among the major downstream profibrogenic mediators of TGF-*β*. Inhibition of the RAS with ACE inhibitors or ARBs is directly correlated with suppression of TGF-*β* production and amelioration of fibrosis [52, 54].

Inflammation may activate the RAS, which in turn may further contribute to vascular remodeling in hypertension. Activators of nuclear receptors, such as the peroxisome proliferator-activated receptors (PPARs), downregulate the vascular inflammatory response in experimental animals [55] and decrease serum markers of inflammation in humans [56]. Hence, PPARs may be endogenous modulators of the inflammatory process involved in vascular structural changes in hypertension. On the other hand, Ang II downregulates PPARs through activation of NF-*κ*B [57].

Ang II may also play a role in EPC production. This effect may be dependent on the duration of Ang II exposure [58]. Acute Ang II administration via AT_1_R synergistically increases vascular endothelial growth factor-(VEGF-) induced proliferation of EPCs [59]. Moreover, in Ang II-infused mice or in a clipped kidney mouse model of hypertension, the number of circulating EPCs increased. This may be dependent on an increase in cell proliferation as well as on a reduction in apoptosis via Akt and eNOS-derived NO production [60]. AT_1_R antagonism blocked this effect [61]. On the other hand, it has been also shown that Ang II decreases EPC number. Infusion of Ang II in rats is associated with decreased EPC number that is reversible by treatment with an ARB [62]. AT_1_R blockade increased EPC numbers in type 2 diabetic patients [5]. Infusion of Ang II in rats significantly reduced telomerase activity and accelerated the onset of cellular senescence in EPCs, suggesting that reduction in EPC levels after Ang II infusion results from impaired EPC proliferation. Studies in cultured EPCs suggest that the mechanism of Ang II-mediated EPC senescence may also involve ROS [63]. In these cells, the induction of EPC senescence was found to be associated with NADPH oxidase-dependent generation of ROS. Indeed, treatment with superoxide dismutase (SOD) blocked Ang II-mediated EPC senescence.

ROS are potent stimulators of endothelin-1 synthesis by endothelial cells and VSMCs [64]. Endothelin-1 in turn activates NADPH oxidase as well as other sources of ROS, such as xanthine oxidase and mitochondria, in VSMCs and blood vessels [65–67]. Moreover, Ang II-induced inflammation via NF-*κ*B and AP-1 activation involves in part endothelin receptors [68]. Endothelin-1-induced oxidative stress promotes inflammatory responses and in turn contributes to the vascular remodeling and endothelial dysfunction in animal models of hypertension that present an endothelin-mediated component [69]. ET_A_ (Endothelin A) receptor antagonism decreases oxidative stress, normalizes hypertrophic remodeling, decreases collagen and fibronectin deposition, and reduces adhesion molecules in the vasculature of aldosterone-infused rats [70]. Endothelial dysfunction and increased activity of NADPH oxidase were described [68] in mice overexpressing preproendothelin-1 in the endothelium. This was associated with enhanced oxidative stress and vascular inflammation [68] and, in turn, to increased arterial stiffness as well as to increased collagen deposition, independently of blood pressure [68]. In humans, exogenous endothelin-1 has also been shown to increase arterial stiffness [71, 72]. Hence, in endothelial dysfunction where NO is downregulated and endothelin-1 upregulated, the balance is shifted in favor of increased arterial stiffness.

Ang II induces aldosterone synthesis which in turn increases tissue ACE activity [73] and up-regulates angiotensin receptors [74]. The activation of mineralocorticoid receptors may contribute to cardiovascular dysfunction, inflammation, fibrosis, and vascular damage. Several animal models have confirmed that aldosterone may induce ROS formation and endothelial dysfunction, and therefore it can cause injury of the vasculature of the brain, heart, and kidneys [75]. Endothelial dysfunction and inflammation induced by aldosterone may involve the activation of COX-2 (cyclooxygenase-2) in normotensive and hypertensive rats [76]. Mineralocorticoid antagonism attenuates the aldosterone-induced damage by reducing the direct proinflammatory and pro-fibrotic effects of aldosterone that may be mediated via activation of the endothelin system [70, 77–79]. Mineralocorticoid receptor blockade may also improve endothelial function and reduce oxidative stress in Ang II-infused rats [80]. This suggests that aldosterone may induce in part actions usually attributed to direct effects of Ang II in the vasculature.

## 4. New Biomarkers of Vascular Damage

Several studies indicate that the microparticles of endothelial origin may be considered as biomarkers of vascular dysfunction. Microparticles are small vesicular fragments (<1 *μ*m) of cellular membranes derived from most cell types including endothelial and inflammatory cells. They are released in response to activation, injury, and apoptosis [81]. Microparticles circulate in the plasma of healthy individuals, and their levels increase in cardiovascular and atherothrombotic diseases [82]. Microparticles may contribute to vascular disease progression in hypertension. In patients with severe hypertension plasma levels of microparticles correlated with systolic and diastolic blood pressure [83]. In patients with diabetes, endothelial microparticle levels are a strong predictor of myocardial infarction and correlate with arterial stiffness and endothelium-mediated vasodilation [84, 85]. They also relate to the extent and severity of coronary stenosis in patients with coronary syndromes [86]. In vitro endothelial microparticles are released in response to inflammatory stimuli [87] (including TNF-*α*, thrombin, uremic toxins, ROS, and PAI-1). Although the precise mechanism leading to generation of microparticles from endothelial cells is not fully understood, there are pieces of evidences that endothelial NO synthase uncoupling and low shear stress as well [88, 89] enhance their production. Microparticles in turn may contribute in part to endothelial dysfunction itself through the activation of ROS production [90, 91]. Microparticles are capable of impairing vasorelaxation. They interfere with target cell responses by transferring chemokines and adhesion molecules to endothelial cells leading to monocyte adhesion [92] and therefore contributing to vascular injury in hypertension. Ang II may induce microparticle formation, although the evidences are controversial. Ang II type 1 receptor (AT_1_R) blockade is associated with a reduction in the number of monocyte, platelet, and endothelial cell-derived microparticles in hypertensive diabetic patients [93–95]. However Ang II administration in cultured HUVEC did not result in an increased microparticles formation [96].

## 5. Conclusion

Functional and structural alterations of resistance arteries are the earliest vascular alterations that may occur in hypertension. Low-grade inflammation and endothelial dysfunction are strictly associated in the development of hypertension and its complications. The activation of RAS plays a key role in the genesis of endothelial dysfunction and vascular remodeling. Ang II activates redox-sensitive pathways and promotes cell growth and inflammation, involving, in part, factors derived from microparticles and EPCs. Antihypertensive agents have been shown to partially correct the vascular remodeling and impaired endothelial function particularly of small resistance arteries in both experimental models and human hypertension. In particular antihypertensive drugs that antagonize RAS, including ACE inhibitors, ARBs, mineralocorticoid receptor antagonists, and the renin inhibitors, embody valid therapeutic tools to improve vascular function and reduce vascular remodeling and cardiovascular risk.
